# Neuroprotective Effect of β-Caryophyllene on Cerebral Ischemia-Reperfusion Injury via Regulation of Necroptotic Neuronal Death and Inflammation: *In Vivo* and *in Vitro*

**DOI:** 10.3389/fnins.2017.00583

**Published:** 2017-10-26

**Authors:** Mei Yang, Yongjiu Lv, Xiaocui Tian, Jie Lou, Ruidi An, Qian Zhang, Minghang Li, Lu Xu, Zhi Dong

**Affiliations:** ^1^Chongqing Key Laboratory of Biochemistry and Molecular Pharmacology, School of Pharmacy, Chongqing Medical University, Chongqing, China; ^2^Chongqing Research Center for Pharmaceutical Engineering, School of Pharmacy, Chongqing Medical University, Chongqing, China; ^3^School of Pharmacy, Chongqing Medical and Pharmaceutical College, Chongqing, China

**Keywords:** necroptosis, inflammation, β-caryophyllene, ischemia-reperfusion injury, oxygen-glucose deprivation and re-oxygenation, high-mobility group box 1

## Abstract

Necrotic cell death is a hallmark feature of ischemic stroke and it may facilitate inflammation by releasing intracellular components after cell-membrane rupture. Previous studies reported that β-caryophyllene (BCP) mitigates cerebral ischemia-reperfusion (I/R) injury, but the underlying mechanism remains unclear. We explored whether BCP exerts a neuroprotective effect in cerebral I/R injury through inhibiting necroptotic cell death and inflammation. Primary neurons with and without BCP (0.2, 1, 5, 25 μM) treatment were exposed to oxygen-glucose deprivation and re-oxygenation (OGD/R). Neuron damage, neuronal death type and mixed lineage kinase domain-like (MLKL) protein expression were assessed 48 h after OGD/R. Furthermore, mice underwent I/R procedures with or without BCP (8, 24, 72 mg/kg, ip.). Neurologic dysfunction, cerebral infarct volumes, cell death, cytokine levels, necroptosis core molecules, and HMGB1-TLR4 signaling were determined at 48 h after I/R. BCP (5 μM) significantly reduced necroptotic neurons and MLKL protein expression following OGD/R. BCP (24, 72 mg/kg, ip.) reduced infarct volumes, neuronal necrosis, receptor-interaction protein kinase-1 (RIPK1), receptor-interaction protein kinase-3 (RIPK3) expression, and MLKL phosphorylation after I/R injury. BCP also decreased high-mobility group box 1 (HMGB1), toll-like receptor 4 (TLR4), interleukin-1β (IL-1β), and tumor necrosis factor-α (TNF-α) levels. Thus, BCP alleviates ischemic brain damage potentially by inhibiting necroptotic neuronal death and inflammatory response. This study suggests a novel application for BCP as a neuroprotective agent.

## Introduction

Stroke is a common cerebrovascular disease associated with high disability and mortality worldwide, and ~85% of strokes are caused by cerebral ischemia (Flynn et al., 2008). To date, tissue plasminogen activator (tPA) is an effective therapeutic agent that has been approved by the FDA for ischemic stroke. However, its severe side effects and limited timeframe for administration make it applicable only for 4–7% of all stroke patients (Tobin et al., 2014). Thus, novel effective therapeutic strategies are required.

Necrotic cell death is a hallmark feature of ischemic stroke, yet lacks therapeutics to specifically target necrosis because it has long been characterized as an unregulated and irreversible cell death. Recent studies suggested that there is a form of regulated and programmed necrosis, called necroptosis (Degterev et al., 2005; Vandenabeele et al., 2010). Necroptosis was thus named because it possesses many of the morphological characteristics of accidental necrosis but manifests some important inductive-phase features of apoptosis. The best-described necroptosis process requires receptor-interacting protein kinase 1 (RIPK1), receptor-interacting protein kinase 3 (RIPK3) (Cho et al., 2009; He et al., 2009; Zhang et al., 2009), and its substrate, the mixed lineage kinase domain-like protein (MLKL) (Hsu et al., 1996; Holler et al., 2000; Sun et al., 2012; Wang H. et al., 2014). RIPK3-mediated phosphorylation of MLKL promotes its oligomerization that translocated spontaneously to the nuclei and plasma membranes, which induces necroptotic cell death (Cai et al., 2014; Chen et al., 2014; Yoon et al., 2016). Necrostain-1 (Nec-1), a specific small-molecule inhibitor of RIPK1, can inhibit necroptosis through blocking the formation of necrosome and phosphor-MLKL signal (Wang H. et al., 2014). Nec-1 has been used to ameliorate tissue injury in murine models of ischemia-reperfusion (I/R) in the brain (Degterev et al., 2005; Xu et al., 2010; Yin et al., 2015), which revealed that necroptosis is involved in brain injury caused by I/R and may serve as a putative therapeutic target to treat cerebral I/R injury.

Necroptotic cell death may promote inflammation by releasing intracellular components and damage-associated molecular patterns (DAMPs) after plasma membrane rupture (Kaczmarek et al., 2013). High-mobility group box 1 protein (HMGB1), released passively from necrotic or injured cells, functions as a DAMPs to induce inflammatory response (Vogel et al., 2015). The pro-inflammatory roles of HMGB1 is mediated by cell surface receptors including Toll-like receptor (TLR)4, TLR2, and receptor for advanced glycation end products (RAGE) (Yu et al., 2006; Weber et al., 2015; Li et al., 2016). In CNS, HMGB1 is ubiquitously expressed in neurons at high levels (Frank et al., 2015). Inhibition of HMGB1 signaling reduced infarct volume, microglia activation, and neuro-inflammation in murine model of cerebral ischemia (Liu et al., 2007; Liesz et al., 2015). Additionally, numerous studies showed that TLR4 plays a crucial role in I/R injury by recognizing and binding to HMGB1, with a resulting inflammatory response (Li et al., 2006; Yang et al., 2010).

β-caryophyllene (BCP), a bicyclic sesquiterpene that extracted from many essential oils of food plants and spices, displays diverse biological and pharmacological activities, such as, anti-inflammatory (Cho et al., 2007; Horvath et al., 2012; Chang et al., 2013), anticancer (Kim et al., 2014), and antianxiety properties (Galdino et al., 2012; Bahi et al., 2014). BCP alleviates D-galactosamine- and lipopolysaccharide-induced liver injury by suppressing TLR4-mediated inflammatory responses (Cho et al., 2015). In addition, previous researches indicated that BCP could reduce infarct volume and alleviate ischemic neuron injury in cerebral ischemia (Chang et al., 2013; Lou et al., 2016). However, the mechanisms by which BCP confers neuroprotection remain elusive. In this study, we investigated the effect of BCP on necroptotic cell death and neuro-inflammation mediated by HMGB1/TLR4 signaling in cerebral I/R model *in vivo* and *in vitro*.

## Materials and methods

### Materials

β-caryophyllene, 2,3,5-triphenyltetrazolium chloride (TTC), propidium iodide (PI), Hoechst 33342, cytosine arabinoside (Ara-C), L-glutamine, and poly-L-lysine (0.1%) were purchased from Sigma (Sigma-Aldrich, St. Louis, MO, USA). All cell culture medium and fetal bovine serum (FBS) were obtained from GIBCO (Life Technologies, Grand Island, NY, USA). The Annexin V-FITC Apoptosis Detection Kit and TUNEL FITC Apoptosis Detection Kit were purchased from Vazyme (Vazyme Biotech Co., Ltd, Nanjing, China). ELISA kit against interleukin-1β (IL-1β), tumor necrosis factor-α (TNF-α) were obtained from USCN (Life Science Inc., Harrington Oakland, CA, USA). Lactate dehydrogenase (LDH) was purchased from Nanjing Jiancheng company (Nanjing, China).

### Animals

Pregnant C57BL/6 mice (embryonic day 17–19) and adult male mice C57BL/6 (20–25 g) in a specific pathogen-free (SPF) grade were obtained from the Experimental Animal Center, Chongqing Medical University (Chongqing, China). All animal procedures were approved by the Experimental Ethics Committee of Chongqing Medical University (Reference Number: 2015027) and performed in accordance with the National Institutes of Health Guide for the Care and Use of Laboratory Animals. All surgeries were performed under anesthesia, and all efforts were made to minimize the animals' suffering.

### Primary neuron cultures

Primary hippocampal neurons were prepared from mice embryos (17–19 days gestation) as described previously (Zhang et al., 2007). Hippocampi were minced and dissected with trypsin-EDTA (0.125 mg/mL) in Hank's balanced salt solution (HBSS). Neurons were cultured in plates, pre-coated with 0.01%poly-L-lysine, with Dulbecco's Modified Eagle's Medium (DMEM) medium containing 10% FBS. After 4 h of incubation, neurons were maintained in neurobasal A medium supplemented with 2% B27 and 1% glutamine (2 mM). Every 3 days, 50% of the culture medium was changed. Microtubule-associated protein-2 (MAP 2), the specific marker of neuron, was used to identify the purity of primary neurons by immunofluorescence. The primary cells, which commonly consist of >95% neurons, were used in the experiments on the 7th day *in vitro*.

### Oxygen-glucose deprivation and re-oxygenation and BCP treatment

Oxygen-glucose deprivation and re-oxygenation (OGD/R) were used as an *in vitro* model for ischemia, as previously described with slight modifications (Zhang et al., 2007; Vieira et al., 2014). Briefly, at the seventh *in vitro*, neurons were washed and incubated with glucose-free medium, subsequently transferred to an anaerobic incubator equilibrated with 94% N_2_, 5% CO_2_, and 1% O_2_ at 37°C for 2 h. The cells were then returned to the anormoxic incubator with 25 mM glucose without serum for 48 h. Control neurons were cultured in the same medium supplemented with 25 mM glucose in a normoxic incubator.

BCP (Sigma-Aldrich, St. Louis, MO, USA) and Nec-1 were prepared in 0.05% dimethyl sulfoxide. Nec-1 (10 Mm; Degterev et al., 2005) and BCP (0.2, 1, 5, 25 μM) were applied for 1 h before OGD/R and maintained throughout OGD/R. Neurons were randomly separated into seven groups: (i) the control group, incubated in cell culture medium containing 0.05% dimethyl sulfoxide alone; (ii) the OGD/R group, which was exposed to OGD/R; (iii) the BCP (0.2 μM) + OGD/R group; (iv) the BCP (1 μM) + OGD/R group; (v) the BCP (5 μM) + OGD/R group; (vi) the BCP (25 μM) + OGD/R group; and (vii) the Nec-1 (10 μM)+OGD/R group.

### Cell damage and death assays

Neuron damage was determined by LDH leakage assay at 48 h of recovery following the OGD insult. Extracellular LDH released from damaged neurons and intracellular LDH were measured after the cells were lysed using an LDH detection kit, according to the manufacturer's instructions. The leakage rate of LDH was expressed as a percentage of LDH released in the culture medium/total LDH (media and lysates).

Neuronal death was evaluated with the nuclear morphology analysis at the indicated time. Primary neurons were stained with both the nuclear dyes PI (5 μg/mL)/Hoechst 33342 (2 μg/mL) for 15 min at 37°C, and observed immediately using fluorescence microscopy (Eclipse Ti-S, Nikon, Japan). Ten random fields were captured per well and the cells were counted in each field. The results were presented relative to necrotic cell death (positive PI but no pyknosis/total neuron number) or the percent of apoptotic-like cell death (pyknotic nuclei/total neuron number). For annexin V/PI staining, primary neurons were incubated with annexin V and PI, according to the manufacturer's instructions. Necrotic cell death was confirmed by AnnexinV/PI double positivity and PI single positivity, in the absence of cells with AnnexinV single positivity.

### Immunofluorescence staining

At 48 h after OGD/R, 4% paraformaldehyde was used to fix primary neurons for 10 min. Following fixation, primary neurons were incubated with MAP-2 (4542, Cell Signaling Technology, USA, 1:100) or MLKL (21066-1-AP, Proteintect, China, 1:50) antibodies overnight at 4°C, and then incubated with FITC-goat anti-rabbit IgG (A23220-1, Abbkine, USA, 1:200) or cy3-conjugated goat anti-rabbit IgG (SA00009-2, Proteintech, 1:200). Then, 4′,6-diamidino-2-phenylindole (DAPI, C1006, Beyotime, China) was used to stain the nuclei. These stained sections and primary neurons were observed with a fluorescence microscope and confocal laser scanning microscope (CLSM, Nikon A1R, Japan), respectively.

### Transient focal cerebral ischemia and BCP treatment

Male mice underwent procedures to cause transient focal cerebral ischemia via right middle cerebral artery occlusion (MCAO), as previously described (Sugo et al., 2002). Briefly, mice were anesthetized with isoflurane (induced with 3% and maintained by 1.0–1.5%) mixed with oxygen and nitrogen using a facemask. The right common carotid artery (CCA), internal carotid artery (ICA), and external carotid artery (ECA) were separated carefully under an operating microscope. A 6-0 nylon monofilament (Guangzhou Jialing Biotechnology Co., Ltd., Guangzhou, China) was inserted through the stump of ECA into the ICA and advanced into the middle cerebral artery until light resistance was felt (~8–12 mm). After 1 h of MCAO, reperfusion was initiated by withdrawing the nylon monofilament. Sham-operated mice underwent identical procedure but the filament was not inserted. During the surgical procedure, rectal temperature was maintained at 37 ± 5°C using a thermostatically controlled infrared lamp. At 48 h of reperfusion, neurological function deficits were scored, and animals that scored from 1 to 4 were chosen for further experiment. Those animals that showed brain hemorrhage or with no ischemia (three mice) were exclude from the study. The mortality rate was 0.3%.

Male C57BL/6 mice were divided randomly into five groups: sham, I/R, I/R + BCP (8 mg/kg), I/R + BCP (24 mg/kg), and I/R + BCP (72 mg/kg). BCP solution was prepared in olive oil (Horvath et al., 2012; Chang et al., 2013; Al Mansouri et al., 2014). For the BCP+I/R group, mice were administered intraperitoneally once daily for 3 consecutive days before undergoing ischemia, and 2 h after focal ischemia. The sham and I/R groups were also treated with vehicle (olive oil) only.

### Evaluation of ischemic outcomes

Ischemic degrees were determined according to neurologic dysfunction and cerebral infarct volume. At 24 h after inducing ischemia, neurologic dysfunction was scored by a blinded observer using the modified Longa's method (Longa et al., 1989). For quantification of cerebral infarct volume, mice in each group were sacrificed, and the mouse brains were quickly isolated, frozen, and cut into four coronal sections with 1-mm thickness. The brain sections (1-mm) were incubated in 2% TTC at 37°C for 15 min, and transferred to 4% paraformaldehyde overnight. The infarct volume was analyzed quantitatively using Image-pro Plus software and expressed as percentage of contralateral hemisphere.

### Immunofluorescence of cleaved caspase-3 and tunel staining

At 48 h after I/R, mice were deeply anesthetized and transcardially perfused with saline followed by 4% paraformaldehyde (0.01 M PBS, PH 7.4), then mouse brains were taken and post-fixed in 4% paraformaldehyde for 12 h. Brain frozen sections in the coronal plane were sliced (5 μm thickness). The co-staining of TUNEL and cleaved caspase-3 in brain sections were performed using immunofluorescence detection of cleaved caspase-3 and using a TUNEL FITC Apoptosis Detection Kit. Briefly, the brain frozen sections (5 μm) were fixed in acetone for 10 min at room temperature. After three washing with 0.1 M PBS, the sections were incubated with proteinase K (20 μg/mL) for 5 min, followed by blocking with 10% goat serum. Sections were then incubated with rabbit polyclonal antibody against cleaved caspase-3 (9661, Cell Signaling Technology, 1:100) overnight at 4°C. After incubating with 1X equilibration buffer for 15 min, the sections were then incubated with Alexa Fluor cy3-conjugated goat anti-rabbit IgG supplemented with TdT according to the manufacturer's instructions. The TUNEL-positive and cleaved caspase-3-negative cells in each specimen were analyzed with a fluorescence microscope.

### Hematoxylin and eosin

Mice brains were infused with 4% neutral-buffered formaldehyde at indicated time, fixated for 24 h. Ethanol in graded concentrations and xylene were then used to dehydrate the brain tissue, and then they were embedded into paraffin. Hematoxylin and eosin (H&E) were used to stain the paraffin sections (5 μm), according to the standard protocol. Histological analysis of the same region in each experiment was performed with a light microscope.

### Transmission electron microscopy

Ultrastructure of ischemic brain tissue was analyzed using transmission electron microscopy (TEM). Mice were deeply anesthetized and then fixed with 4% paraformaldehyde containing 1% glutaraldehyde by perfusion. The hippocampus CA1 region was sliced into cubes (1 mm^3^), and then fixed in glutaraldehyde (2.5%), and osmic acid (1%) for 4 h, respectively. Specimens were dehydrated with acetone and embedded by Epon812. The ultrathin sections of the cubes were dyed with uranyl acetate and lead citrate, and then observed under TEM (Hitachi 7100, Japan).

### Enzyme-linked immunosorbent assay (ELISA)

TNF-α, IL-1β levels in ischemic brain tissue homogenate were detected using an ELISA kit according the manufacturer's instructions.

### Western blot analysis

Mouse ischemic brain tissues were harvested at 48 h post-reperfusion, and then homogenized in RIPA lysis buffer (P00113D; Beyotime, Shanghai, China). Hippocampus of ischemic brain were used to determine RIPK1, RIPK3, and phosphor-MLKL levels. The whole ischemic brain tissues were used to determine HMGB1 and TLR4 protein levels. The protein samples were quantified with a bicinchoninic acid protein assay kit (P0012S, Beyotime, China). The protein was separated using sodium dodecyl sulfate-polyacrylamide gel electrophoresis (SDS-PAGE; P0012A, Beyotime, China) with a 12% polyacrylamide gel, and then transferred to polyvinylidene fluoride (PVDF) membrane. Then the membranes were blocked with non-fat milk (5%) and incubated overnight at 4°C with the following primary antibodies: rabbit polyclonal antibody against RIPK1 (3493, Cell Signaling Technology, 1:1,000), RIPK3 (ab62344, Abcam, 1:1,000), HMGB1 (10829-1-AP, Proteintech, 1:250), TLR4 (19811-1-AP, Proteintech, 1:250), MLKL phosphor S345 (ab196436, Abcam, 1:1000), and mouse polyclonal antibody against glyceraldehyde-3-phosphate dehydrogenase (GAPDH; SC-32233, Santa Cruz, USA, 1:500). After three washes, secondary goat anti-rabbit/mouse (Bostor, China, 1:3,000) was performed to conjugate with alkaline phosphatase for 1 h at room temperature. Enhanced chemiluminescence was used to determine the immune reactivity. Gel imaging apparatus (Bio-Rad, Hercules, CA, USA) and Image Lab (Bio-Rad, Hercules, CA, USA) were used to scan and analyze the bands.

### Real time quantitative polymerase chain reaction analysis

Total RNA of hippocampus of ischemic brain were extracted using a Trizol regent kit (TaKaRa Bio Inc., Japan) and cDNA was prepared via reverse-transcription using the PrimeScript™ RT Reagent Kit with gDNA Eraser (TaKaRa Bio Inc.), according to manufacturer's protocol. Real-time quantitative polymerase chain reaction (RT-qPCR) was performed in a 10 μL volume using SYBR Premix Ex Taq™ II (TaKaRa Bio Inc.). The following cycling conditions were used: 30 s at 95°C followed by 40 cycles of 5 s at 95°C and 30 s at 60°C. The specific primers are listed as Table 1. Gene expression was determined relative to the housekeeping gene GAPDH using the 2^−ΔΔCt^ method.

**Table 1:** Primer sequences.

**Gene**	**Sequence**
RIPK1	Forward 5′-GCAGGAGCAAGAGGTCATTC-3′
	Reverse 5′-TGGCTTAGATTTGGCGGATA-3′
RIPK3	Forward 5′-GGCTCTCGTCTTCAACAACTG-3′
	Reverse 5′-CCGAACTGTGCTTGGTCATA-3′
MLKL	Forward 5′-CCCATTTGAAGGCTGTGATT-3′
	Reverse 5′-ATGATTTCCCGCAACAACTC-3′
GAPDH	Forward 5′-CGTCTTCACCACCATGGAGAAGGC-3′
	Reverse 5′-AAGGCCATGCCAGTGAGCTTC CC-3′

### Statistical analysis

All data are presented as the means ± SEM. SPSS v13 software was used for statistical analysis. Comparisons among different groups were performed using a one-way analysis of variance (ANOVA) followed by Tukey's test. A value of *P* < 0.05 was defined as statistically significant.

## Results

### BCP reduces necrotic neuron death induced by OGD/R

**Figure 1:**
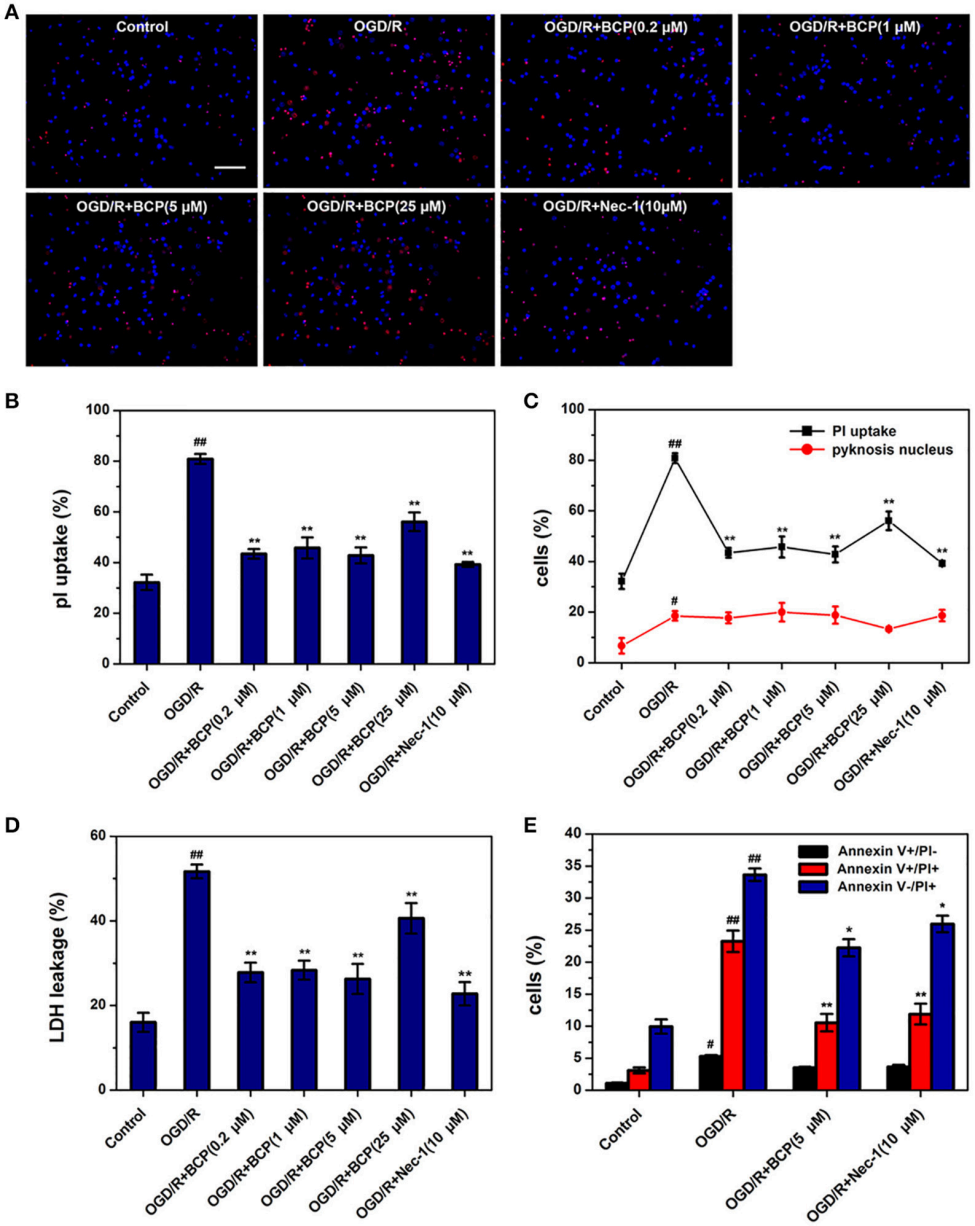
BCP reduces necrotic cell death induced by OGD/R in primary hippocampal neuron culture. Neuronal death was performed at 48 h after OGD in different groups. **(A)** Representative fluorescence micrographs of PI and Hoechst staining of primary neurons exposed to OGD/R. Scale bar 300 μm. **(B)** Quantitative analysis of necrotic neurons (PI-positive cells without nuclear condensation). **(C)** Comparison of necrotic neurons and apoptotic-like neurons (featured by nuclear pyknosis) following 1 h OGD and 48 h re-oxygenation. **(D)** LDH release analysis. **(E)** Quantitative analysis of annexin V and PI staining. Data are presented as the mean ± SEM (*n* = 6). ^#^*p* < 0.05, and ^##^*p* < 0.01 *vs*. sham; ^*^p < 0.05, and ^**^p < 0.01 *vs*. I/R group.

To investigate whether ischemic neuronal death occurs via necroptosis after cerebral ischemia *in vitro*, OGD/R-induced neuronal death was measured by different cell death assays. As shown in Figure 1, necrotic cells and LDH release of primary cultured neurons were significantly increased in the OGD group, while the amounts of apoptotic cells did not show significant difference. Additionally, Nec-1, which inhibits necroptosis, reduced increased necrotic cells and LDH leakage rate after OGD/R injury from 80.84 ± 2.00 and 51.69 ± 1.62% to 39.24 ± 0.96 and 22.76 ± 2.75%, indicating that necroptotic neuronal death occurs in primary neurons following ischemia *in vitro*. BCP (0.2, 1, 5 μM) remarkably reduced the amounts of necrotic cells that had PI positive nuclei (42.79 ± 3.16%), but pyknosis was not present, as it was for Nec-1 (Figures 1A–C). However, BCP had no remarkable effect on apoptotic cells (featured by pyknosis, Figures 1A,C). BCP significantly decreased LDH release rate (26.24 ± 3.55%, Figure 1D). LDH release indirectly measures if the membrane is leaking. Consistent with PI/Hoechst staining results, BCP (5 μM) had a prominent effect on necrotic neurons but not on apoptotic neurons (Figure 1E). In the OGD/R group, the cells with Annexin V^+^/PI^+^, Annexin V^+^/PI^−^, and Annexin V^−^/PI^+^ were 23.25 ± 1.66, 5.27 ± 0.21, and 33.63 ± 0.98%, respectively. Compared to OGD/R group, BCP treatment decreased the necrotic cells to 10.54 ± 1.36 and 22.24 ± 1.33%. These results suggest that BCP has a significant neuroprotective effect in necrotic neuronal death induced by OGD/R injury.

### BCP inhibits necroptotic neuronal death by down-regulation of MLKL *in vitro*

**Figure 2:**
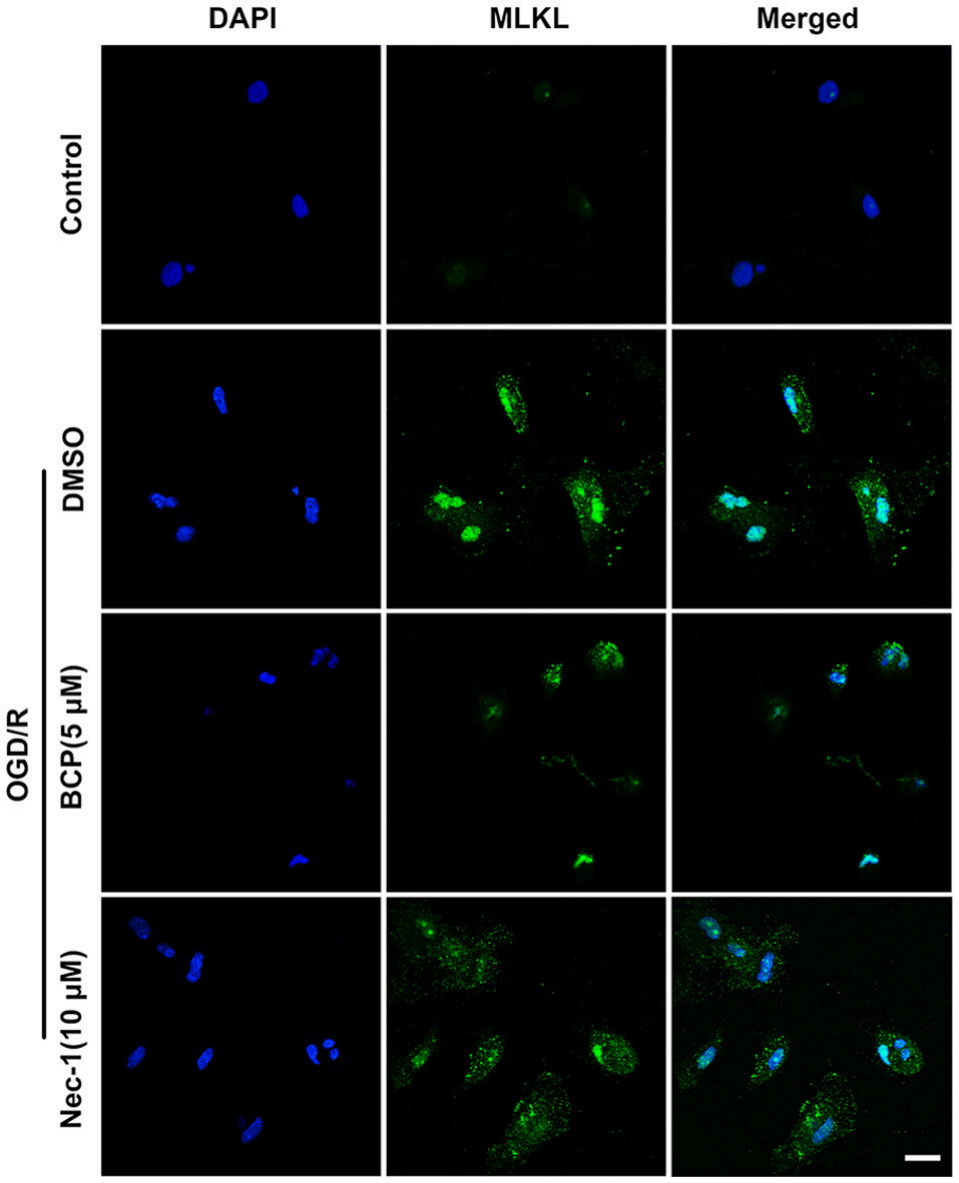
BCP inhibits necroptotic neuronal death by down-regulating MLKL *in vitro*. Primary hippocampal neurons were challenged with OGD for 2 h on the 7th day of culture. CLSM images of primary hippocampal neurons for MLKL at 48 h after OGD (*n* = 3). Scale bar 20 μm.

The signaling cascade that induces necroptotic neuronal death includes RIPK1, RIPK3, and MLKL activation. It is suggested that MLKL triggers necroptotic cell death via translocating to the nucleus (Yoon et al., 2016). To investigate whether BCP mitigates neuronal injury after ischemia by inhibiting necroptotic cell death *in vitro*, we measured MLKL expression using an immunofluorescent assay. As shown in Figure 2, in the OGD/R group, MLKL expression significantly increased and MLKL translocated to the nucleus compared with the control group. However, incubation of primary cultured neurons with BCP (5 μM) significantly ameliorated the increased expression of MLKL after OGD/R injury, indicating that BCP may exerts a neuroprotective effect on ischemic neuronal injury through inhibiting necroptotic cell death.

### BCP alleviates focal ischemia-induced brain injury in mice

**Figure 3:**
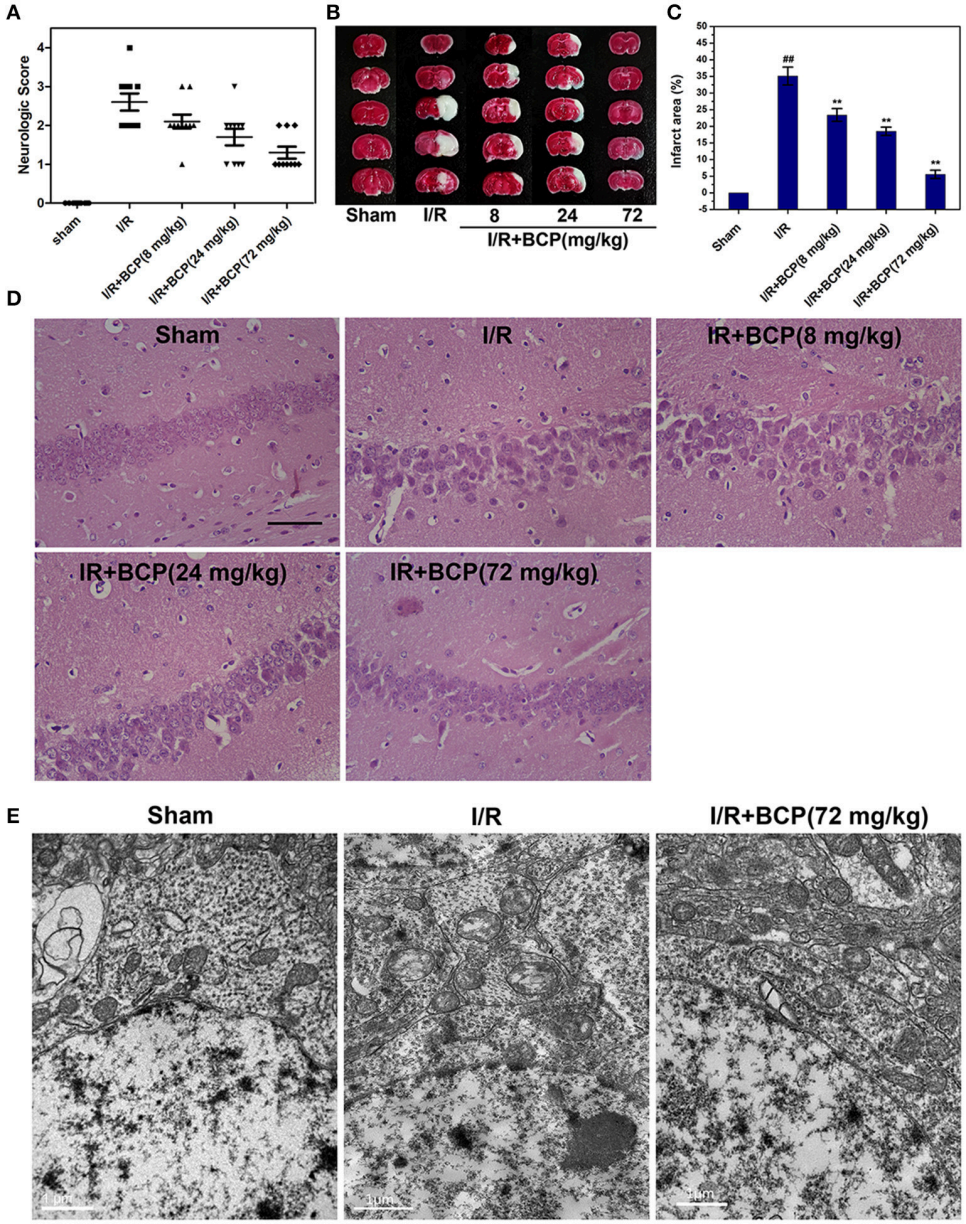
BCP protects the brain against transient focal ischemia in mice. Focal brain ischemia was induced by 60 min of ischemia and 48 h of reperfusion. BCP (8, 24, 72 mg/kg/day) was administered before I/R for three consecutive days. **(A)** Neurological deficit scores of mice subjected to I/R in the five groups (*n* = 10 per group). **(B)** Representative photographs of TTC-stained brain sections of different groups. **(C)** Infarct volumes of mice brain presented as a percentage of intact hemisphere (*n* = 3, ^##^*p* < 0.01 *vs*. sham, and ^**^p < 0.01 *vs*. I/R). **(D)** H&E staining images of the hippocampal CA1 region in brain sections after I/R (*n* = 3). Scale bar 200 μm. **(E)** Representative TEM images of mitochondrial morphologic changes in the hippocampal CA1 region in the mouse brain exposed to I/R (*n* = 3). Scale bar 1 μm.

We examined whether BCP protects the brain against ischemia induced by 48 h of reperfusion after focal ischemia. As shown in Figure 3A, severe neurological deficits were present in the I/R group, while BCP treatment (24 and 72 mg/kg) reduced the neurological score, which suggests that BCP treatment improves the functional outcome after cerebral I/R injury. Additionally, consecutive brain sections stained with TTC were also detected. Compared to the I/R group, BCP (8, 24, 72 mg/kg) treatment decreased the total infarct volume from 35.1 ± 2.68 to 23.4 ± 1.93%, 18.5 ± 1.23, and 5.57 ± 1.25%, respectively (Figures 3B,C), indicating that BCP treatment decreases the brain ischemic area caused by I/R injury.

### BCP inhibits a component of necrotic cell death induced by I/R

To determine the existence of necrotic dying cells in brain subjected to I/R, H&E stained tissues and electron microscopic pictures were analyzed. As shown in Figure 3D, the histological character of the brain in the sham group showed a clear cell outline, orderly arrangement, and compact cell structure. In the I/R group, H&E-stained images displayed pathological alterations in hippocampal CA1 region of injured brains. There were necrotic cells (intact extracellular nuclei) rather than apoptotic cells (apoptotic nuclei appear condensed and fragmented). In contrast, BCP treatment substantially reduced brain damage and improved pathology associated with I/R injury (Figure 3D).

Electron microscopy supplemented with H&E staining was used to illustrate the morphological characteristics of necrotic cells. Neurons showed severe swollen mitochondria in the I/R group, indicating that brain tissue injury in the hippocampal CA1 region induced by I/R, and these neurons mainly underwent necrotic cell death (Figure 3E,). However, BCP treatment (72 mg/kg) significantly alleviated ultrastructural damage on neurons compared with the I/R group.

**Figure 4:**
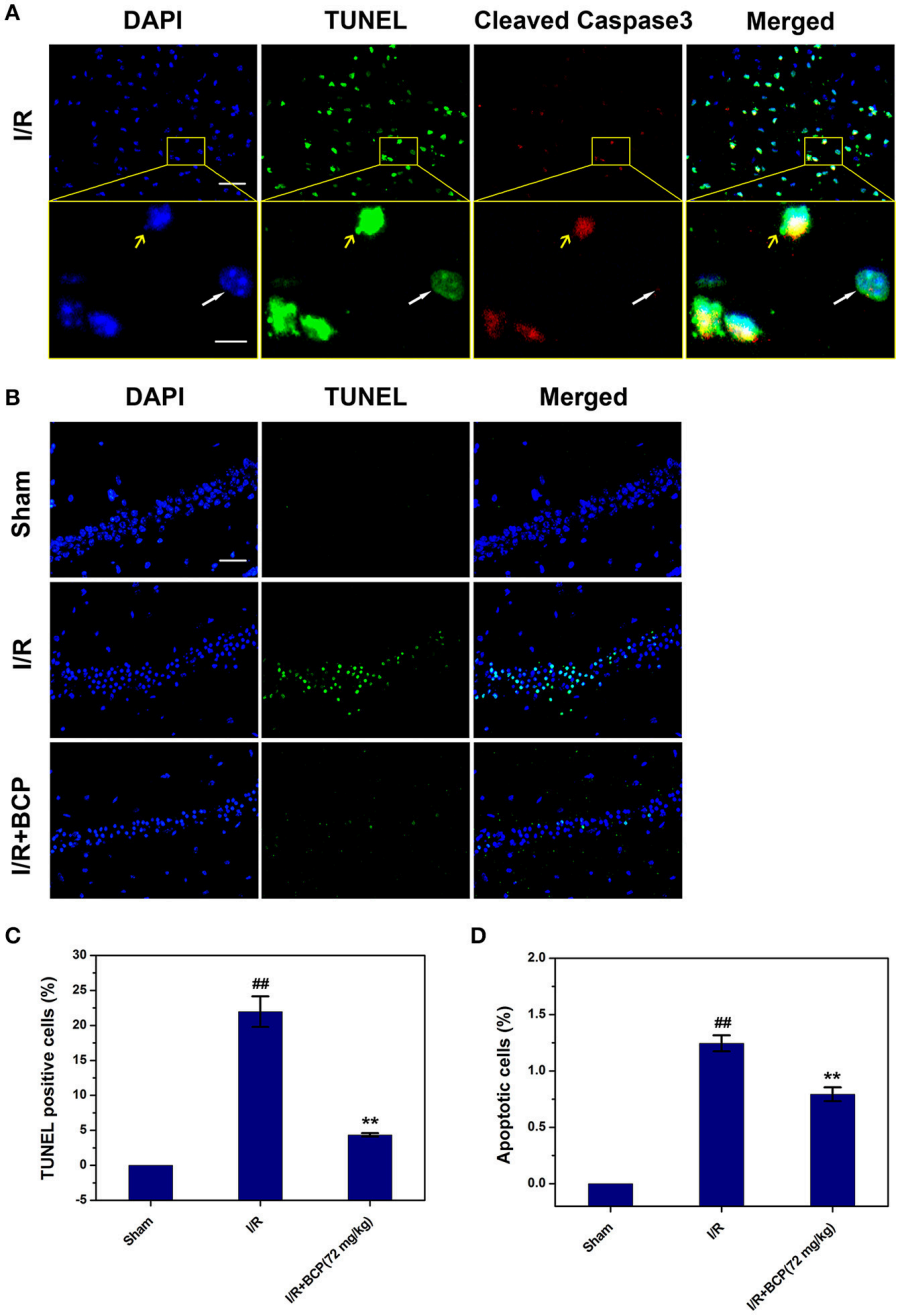
BCP inhibits a component of necrotic cell death induced by I/R. **(A)** Representative microphotographs of TUNEL and cleaved caspase-3-positive cells in the cerebral cortex 48 h after MCAO. Scale bar 100 μm. Areas outlined in yellow are enlarged in lower panels. White arrows show necroptotic cells, and yellow arrows show apoptotic cells. Scale bar 20 μm. **(B)** Representative images of TUNEL-positive cells in the hippocampal CA1 region after I/R in mice treated with or without BCP (72 mg/kg). Scale bar 100 μm. **(C,D)** Percentage of apoptosis (cells with TUNEL and cleaved caspase-3 double positivity) and necrosis (cells with TUNEL-positive showing no cleaved caspase-3 activation) in the mouse brain in response to I/R treatment. Data are presented as the mean ± SEM (*n* = 3). ^##^*p* < 0.01 *vs*. sham, and ^**^p < 0.01 *vs*. I/R.

To further investigate the major cell death type of brain injury induced by I/R, the co-staining of TUNEL and anti-cleaved caspase-3 were used. Many cells were TUNEL-positive in the ischemic area in the I/R group (Figure 4A). In the cerebral cortex, only a few parts of the TUNEL-positive cells displaying pyknotic nuclei were also positive for cleaved caspase-3, and others that lacked apoptosis-like nuclear condensation showed no cleaved caspase-3 activation. In the hippocampal CA1 region, all neurons that were positive with TUNEL revealed no cleaved caspase-3 activation (Figure 3B). These results suggested that the cortex cell death that was caused by I/R injury partly underwent necrosis, and that hippocampal CA1 neuronal death is mainly a form of programmed necrosis. In the I/R + BCP group, the amounts of necrotic cells in the hippocampal CA1 region was significantly decreased compared to the I/R group (Figure 4B). In addition, BCP treatment significantly reduced necroptotic and apoptotic cell death in ischemic brain induced by I/R (from 21.9 ± 2.17 and 1.25 ± 0.07% in the I/R group to 4.35 ± 0.24 and 0.79 ± 0.06% in the 72 mg/kg I/R + BCP group, respectively; Figures 4C,D). Thus, our data indicates that programmed necrotic cell death occupies a larger proportion than apoptosis in mouse brain injury caused by I/R. However, BCP treatment alleviated acute brain injury that was involved in inhibiting necrotic cell death.

### BCP reduces necroptotic cell death by downregulation of RIPK1, RIPK3, and MLKL expression

**Figure 5:**
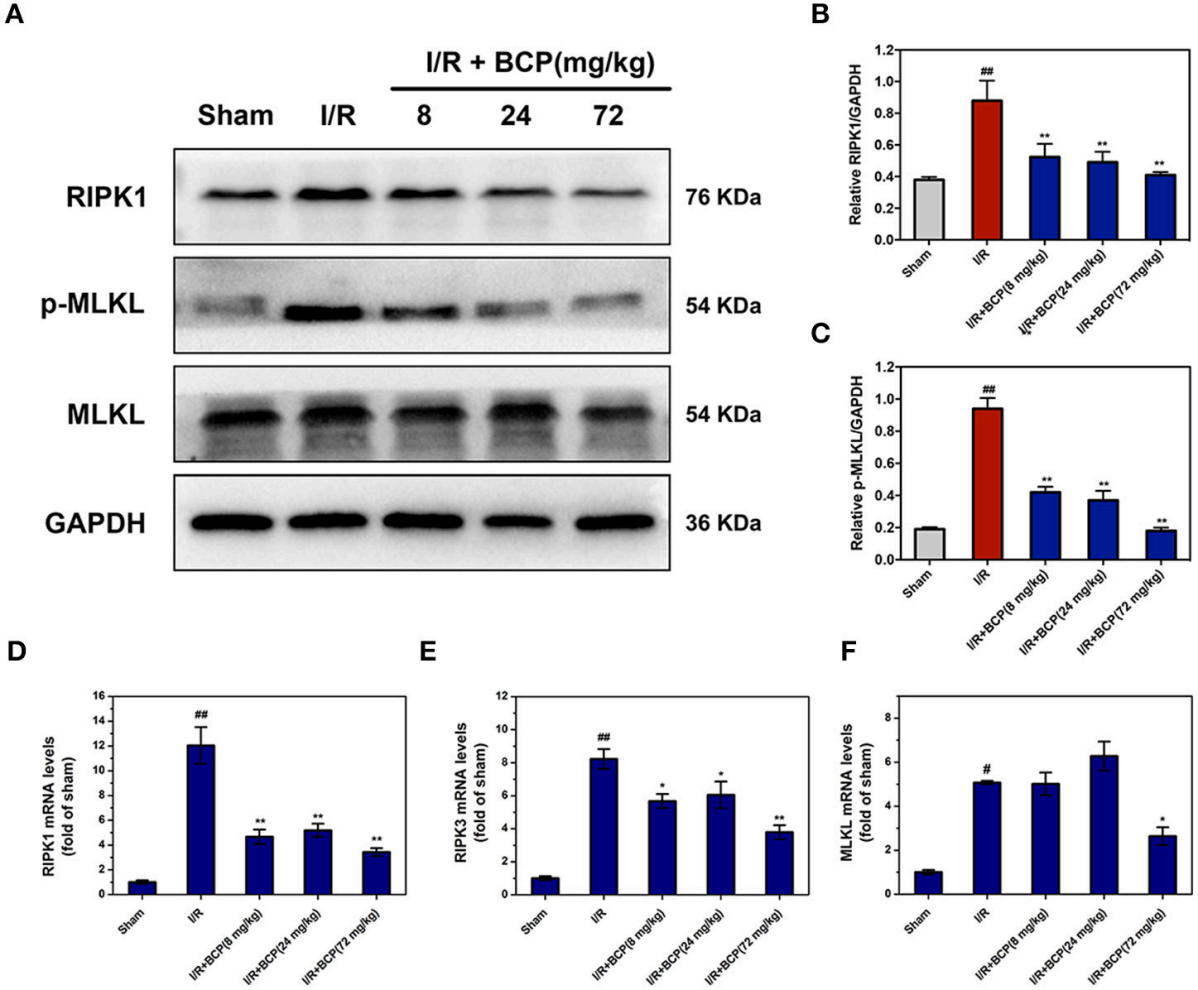
BCP reduces the increased RIPK1, RIPK3 expression and the phosphorylation of MLKL in mouse brains after I/R. **(A)** Representative western blot analysis and RIPK1 **(B)**, p-MLKL **(C)** relative protein expression in the mouse ischemic brain induced by I/R in different groups. Expression is presented relative to GAPDH. RIPK1 **(D)**, RIPK3 **(E)**, and MLKL **(F)** mRNA expression levels in the mouse ischemic brain. Data are presented as the mean ± SEM (*n* = 5) (^#^*p* < 0.05, and ^##^*p* < 0.01 *vs*. sham; ^*^p < 0.05, and ^**^p < 0.01 *vs*. I/R group).

To investigate the necroptosis mechanism by which the cells died *in vivo*, the RIP1, RIP3, and MLKL expression level in the brain were measured using western blot and Q-PCR assay. RIP1 protein levels were significantly increased in the I/R group (Figures 5A,B). RIP3 protein levels, however, did not exhibit a significant change in mice that underwent I/R (data not shown). RIP1, RIP3, and MLKL mRNA levels were significantly upregulated at 48 h after focal ischemia, and BCP treatment effectively reduced levels of this core necroptosis molecule (Figures 5C–E). Additionally, BCP reduced the phosphorylation of MLKL (Figure 5A), suggesting that BCP alleviates ischemic brain injury through inhibiting necroptotic neuronal death.

### BCP alleviates inflammation through inhibiting HMGB1-TLR4 signaling pathway

**Figure 6:**
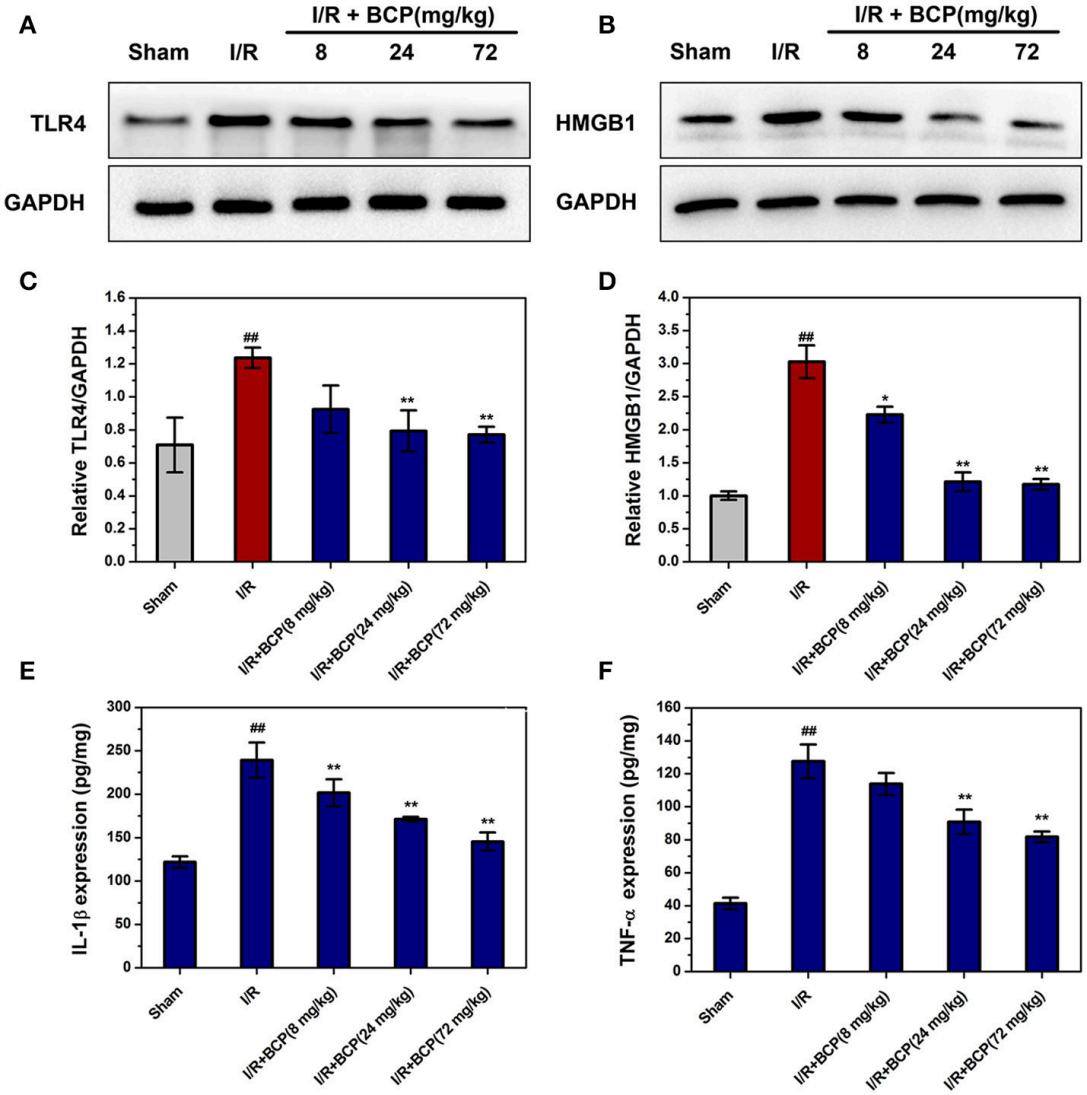
BCP alleviates the inflammatory reaction through suppressing HMGB1-TLR4 signaling. Representative western blot analysis and quantitative analysis of TLR4 **(A,C)** and HMGB1 **(B,D)** in the mouse ischemic brain induced by I/R in the five groups. Expression is presented relative to GAPDH. Data are presented as the mean ± SEM (*n* = 5). TNF-α **(E)** and IL-1β **(F)** protein levels in brain by ELISA assay. Data are presented as the mean ± SEM (*n* = 6) (^##^*p* < 0.01 *vs*. sham; ^*^p < 0.05, and ^**^p < 0.01 *vs*. I/R group).

Necroptosis is characterized by plasma membrane rupture and danger-associated molecular pattern (DAMP) release, which are potent stimuli of inflammation. To explore whether necroptotic cell death could trigger inflammation and BCP treatment could exert anti-inflammatory effects, HMGB1 and TLR4 expression and pro-inflammatory mediator secretion were measured by western blot analysis and ELISA assay. In the I/R group, HMGB1 and TLR4 protein levels increased significantly compared with the sham group (Figure 6). BCP (24 and 72 mg/kg) treatment significantly inhibited TLR4 and HMGB1 release in ischemic brain 48 h after ischemia (Figures 6A–D). TNF-α and IL-1β secretion increased in brain after I/R compared with the sham group. However, BCP administration significantly reduced the increase in TNF-α and IL-1β levels (Figures 6E,F). These data suggested that necroptotic neuronal death in brain at 48 h after ischemia could activate TLR4 by releasing HMGB1 and ultimately facilitate the secretion of inflammatory cytokines, which could promote inflammation. In contrast, BCP attenuated this type of inflammatory reaction.

## Discussion

Necroptosis is a pattern of programmed cell death that is mediated by RIP1 and RIP3, which challenges the conventional notion about necrosis being unregulated and irreversible cell death that is caused by accidental damage. Research has recently confirmed that necroptosis contributes to the pathogenesis of numerous diseases involving cell death and inflammation. Our findings revealed that necroptosis occurs in damage brain induced by cerebral I/R. We report for the first time that BCP exerts neuroprotective effect on necroptotic cell death in cerebral I/R model *in vivo* and *in vitro*. We also showed that BCP can reduce inflammatory response through inhibiting the HMGB1/TLR4 signaling pathway *in vivo*.

Subtypes of programmed cell death have been determined by their distinct morphology. Apoptosis is characterized by cell shrinking, membrane blebbing, chromatin condensation, and formation of apoptotic bodies. Necroptosis, however, is marked by organelle swelling and limited chromatin condensation. Pyroptotic cells generate pyroptotic bodies (Chen et al., 2016). Identification of necroptotic cell death should occur using several diverse methods because of the absence of specific discriminative markers. Typically, H&E staining and TEM were used to illustrate the morphological characteristics of necrotic cells (Gunther et al., 2011). TUNEL staining was previously used to detect DNA strand breaks in apoptotic cells, and can also be used to detect necrosis. Unlike apoptosis, necroptosis is independent of caspase activity. Thus, TUNEL and cleaved caspase-3 staining were commonly used to determine necroptotic cell death (Gunther et al., 2011; Welz et al., 2011). The cells that were positively stained for TUNEL and negatively stained for cleaved caspase-3 are considered to be necroptotic cells. With the above-reported methods, our research indicated that TUNEL-positive neurons induced by I/R show no cleaved caspase-3 activation in the hippocampal CA1 region. We also observed that ultrastructure of damaged neurons show signs of necrosis such as, swollen mitochondrion and lack of chromatic condensation in the nucleus. Our data suggested that neuronal death induced by I/R injury mainly undergo necrosis in the hippocampal CA1 region and BCP treatment could inhibit this type of cell death.

This programmed necrotic cell death is initiated by many triggers including the TNF ligand superfamily, activated TLR3, and TLR4 (He et al., 2011; Kaiser et al., 2013), type I interferons (Robinson et al., 2012), and certain pathogens (Upton et al., 2010; Wang X. et al., 2014). However, the mechanisms that underlie necroptosis in ischemic brain injury remain poorly understood. Many studies have suggested that RIPK1, RIPK3, and MLKL expression levels are correlated with sensitivity to necroptosis (Pasparakis and Vandenabeele, 2015). MLKL, a direct RIP3 substrate, targeted the RIP1-RIP3 complex to form an oligomer and then disrupt membrane integrity in necroptosis-inducing process (Sun et al., 2012; Wang H. et al., 2014; Zhang et al., 2016). Necroptosis has been shown to be a component neuronal death in I/R injury. Most evidence of necroptosis was based on Nec-1's protective effect (Degterev et al., 2005; Smith et al., 2007). We showed that a form of neuronal death is inhibited by Nec-1 in primary cultured neurons exposed to OGD/R. Similar protective effects of Nec-1 are observed for BCP, which reduces necroptotic neuronal death cause by OGD/R. We also showed that BCP treatment reduce increased RIPK1, RIPK3 mRNA expression. Additionally, BCP treatment inhibited the phosphorylation of MLKL at 48 h of reperfusion following focal ischemia *in vivo*. Therefore, BCP has a neuroprotective effect on cerebral I/R injury potentially by inhibiting necroptotic cell death.

An accumulating body of evidence indicated that the protein kinases RIPK1, RIPK3, and MLKL contribute to the inflammatory process by inducing necroptosis (Ofengeim and Yuan, 2013; Chan et al., 2015). Moreover, RIPK3 and MLKL deficiency prevented inflammation and cell death (Welz et al., 2011; Dannappel et al., 2014). Additionally, Necroptosis that is dependent upon RIPK3 and MLKL was also shown to worsen tissue inflammation and injury in an acute pancreatitis mouse model (He et al., 2009). Relatively few studies showed that necroptosis occurs along with the release of HMGB1, and also investigated the role of HMGB1 in necroptosis-induced inflammation (Scaffidi et al., 2002; Yanai et al., 2009; Kaczmarek et al., 2013). We concluded that necroptotic cells may be a crucial factor in triggering inflammation through releasing large amounts of DAMP. TLR4 may mediate many extracellular functions of HMGB1 (Yang et al., 2010) and it is a signaling receptor to activate the innate immune system in I/R injury (Wu et al., 2007; Nystrom et al., 2013). In this study, results showed that BCP treatment reduces increased HMGB1 and TLR4 expression and pro-inflammatory cytokine secretion in brain after I/R, suggesting the anti-inflammatory effect of BCP is associated with HMGB1/TLR4 signaling.

In conclusion, our *in vivo* and *in vitro* data indicated that necroptosis is a form of neuronal death that is caused by cerebral I/R injury. Additionally, BCP alleviates brain damage in cerebral I/R model through inhibiting necroptotic cell death and inflammation. Further study are warranted to determine whether the anti-inflammation of BCP on ischemic stroke through impairing HMGB1 release from necroptotic cells.

## Author contributions

ZD, LX, and MY conceived and designed the experiments; MY and XT conducted the experiment. JL, RA, QZ, and ML provided assistance in experiment performing; MY and YL analyzed the data. MY and YL wrote the manuscript. All authors discussed and commented on the manuscript.

### Conflict of interest statement

The authors declare that the research was conducted in the absence of any commercial or financial relationships that could be construed as a potential conflict of interest.

## Abbreviations

BCP: β-caryophyllene
I/R: ischemia-reperfusion
OGD/R: oxygen-glucose deprivation and re-oxygenation
MLKL: mixed lineage kinase domain-like protein
RIPK1: receptor-interaction protein kinase-1
RIPK3: receptor-interaction protein kinase-3
Nec-1: necrostain-1
TLR4: toll-like receptor 4
HMGB1: high-mobility group box 1
TNF-α: tumor necrosis factor-α
IL-1β: interleukin-1β
tPA: tissue plasminogen activator
TTC: 2,3,5-triphenyltetrazolium chloride
PI: propidium iodide
Ara-C: cytosine arabinoside
FBS: fetal bovine serum
LDH: lactate dehydrogenase
SPF: specific pathogen-free
MAP-2: microtubule-associated protein-2
MCAO: middle cerebral artery occlusion
CCA: common carotid artery
ICA: internal carotid artery
ECA: external carotid artery
DAPI: 4′,6-diamidino-2-phenylindole
H&E: hematoxylin and eosin
TEM: transmission electron microscopy
ELISA: enzyme-linked immunosorbent assay
RT-qPCR: real-time quantitative polymerase chain reaction
SDS-PAGE: sodium dodecyl sulfate–polyacrylamide gel electrophoresis
PVDF: polyvinylidene fluoride
GAPDH: glyceraldehyde-3-phosphate dehydrogenase.
